# Multi-level stakeholder perspectives on barriers and facilitators to engaging older adult cancer survivors in mental health treatment

**DOI:** 10.1186/s12885-026-16396-2

**Published:** 2026-07-03

**Authors:** Rebecca M. Saracino, Ellen Y. Park, Devika R. Jutagir, Leah E. Walsh, Jaime Gilliland, Caraline Demirjian, Kelly McConnell, Christian J. Nelson

**Affiliations:** 1 Department of Psychiatry and Behavioral Sciences, Memorial Sloan Kettering Cancer Center, 633 Third Avenue, 4th Floor, New York, NY 10017 USA; 2 Brookdale Department of Geriatrics and Palliative Medicine, Icahn School of Medicine at Mount Sinai, 1 Gustave L. Levy Place Box 1070, New York, NY 10029 USA

**Keywords:** Cancer survivors, Focus groups, Depression, Anxiety, Mental health treatment, Older adult, Aging

## Abstract

**Background:**

The current study sought to identify the barriers to and facilitators of connecting older adult cancer survivors (OACS) to mental health treatment in cancer survivorship and to better understand the opportunities to improve the likelihood that OACS receive appropriate services in survivorship.

**Methods:**

Four virtual focus groups with 22 stakeholders (i.e., OACS, social workers, and APPs) were conducted. A semi-structured focus group guide queried participants about their experiences of referring and connecting to mental health services during cancer survivorship with a focus on barriers and facilitators. Thematic content analysis guided the generation and interpretation of major and minor study themes from the focus groups.

**Results:**

OACS were 56% female [*n* = 5; mean age = 74 (range: 66–85)]. Providers were 92% female (*n* = 12) and 69% (*n* = 9) had been working in oncology for over ten years. Three major conceptual themes were identified: (1) Care pathway characteristics (i.e., limited referral resources and knowledge); (2) Patient-level characteristics (i.e., motivation, stigma); (3) Perspectives on telehealth (i.e., overall increased access to care but may present challenges for some OACS). These overarching themes included sub-themes of barriers and facilitators to connecting OACS to mental health services.

**Conclusions:**

OACS remain disconnected from mental health services and providers struggle to overcome systemic barriers to getting survivors the care they need. Given the standard of care to screen for depression in cancer survivorship, OACS will continue to be identified as depressed with no clear recourse for intervention. Therefore, future research on how to mitigate barriers and improve existing care pathways to connect OACS to mental health treatment is warranted.

**Supplementary Information:**

The online version contains supplementary material available at https://doi.org/10.1186/s12885-026-16396-2.

## Background

Presently, 64% of cancer survivors are ≥ 65 years old, and that number will increase to 73% by the year 2040 [1]. Up to 35% of these older adult cancer survivors (OACS; ≥65 years, post-cancer treatment) have clinically significant depression compared to approximately 20% among older adults more broadly [2–5]. Cancer survivors are twice as likely to report disabling mental health problems compared to adults without cancer, and those with additional chronic illnesses, such as many older adults, are nearly 6 times as likely [6]. Comorbid mental health problems and medical illness is also associated with poor quality of life and symptom control, pain, social isolation, and premature mortality [7, 8]. Depression can also interfere with the ability to make treatment decisions and adhere to follow-up care [9]. Thus, older adults with depression and medical illness have significantly higher healthcare costs, double their non-depressed peers [10]. In the extreme, OACS are 2.3 times as likely as healthy older adults to die by suicide [11, 12]. Additionally, a cancer diagnosis for an older adult often arises in the context of broader aging-related stressors such as retirement, shifts in social support, bereavement, and additional medical comorbidity and/or loss of independence [8–10, 12], all of which may compound mental health vulnerability in older adults.

In response to the deleterious effects of untreated depression, many accrediting healthcare organizations such as the National Comprehensive Cancer Network (NCCN) and the American Society of Clinical Oncology (ASCO), have developed survivorship care guidelines that require healthcare professionals to screen, evaluate, and provide treatment recommendations for patients presenting with depressive symptoms [13, 14]. However, when older patients with significant symptoms are identified and referred for services, the majority will not receive treatment because they are the least likely of any age group to use mental health services [15–20]. One study revealed that up to 70% of older adults with significant mood disorders were not using mental health services [20]. This research, however, has not been conducted in cancer settings and therefore whether or not it holds true in survivorship care, for example, has yet to be determined. Similarly, even when OACS are interested in depression treatment, they may not receive developmentally appropriate services because there are a lack of interventions specifically for this age group and providers lack specialized training in geriatric mental health in cancer care [17]. Additionally, although the National Cancer Institute and National Institute on Aging recommended giving “substantial attention” to the unique needs of older adults in survivorship [21–23], there is sparse evidence on how to best implement depression care for OACS. Therefore, the potentially unique challenges faced when connecting OACS to mental health services in survivorship care are unclear.

The current study sought to identify the barriers to and facilitators of connecting OACS to treatment in cancer survivorship. The aim of this study was to explore the experiences of three stakeholder groups [oncology social workers (SW); advanced practice providers (APP); and OACS] to better understand the challenges and opportunities in this care setting to improve the likelihood that OACS receive appropriate mental health services.

## Methods

### Study design

A qualitative descriptive design employing focus group discussions with three groups of stakeholders (i.e., SWs, APPs, OACS) was used to elucidate barriers to and facilitators of engaging in mental health treatment during cancer survivorship. This study was performed in line with the principles of the Declaration of Helsinki. Approval was granted by the Internal Review Board of Memorial Sloan Kettering Cancer Center (02/19/2021; #X21-010). Verbal agreement for participation was obtained from all participants. Focus groups and associated data collection took place from 10/29/2021 to 09/08/2022. The reporting of study methods and results adheres to the “Consolidated Criteria for Reporting Qualitative Research” (COREQ) [24].

### Research team

Each focus group was conducted by both a qualitative methods specialist (QMS; JG) and a clinical psychologist (RS). The data analysis team included the QMS, three clinical psychologists (RS, DJ, LW), and one clinical research coordinator (CRC; EP). All study processes were overseen by the QMS, who had over 12 years of experience in the field at the time of analyses. Study participants had no prior relationship with the study team. During informed consent, extra emphasis was placed on the voluntary nature of the study. The rationale of the study was explained to participants, and they were given the opportunity to ask questions or seek clarification on study goals.

### Participant selection, setting, and recruitment

English-speaking social work and APP providers working in the Memorial Sloan Kettering Cancer Center (MSK) Survivorship Clinics were contacted internally at MSK by the study Principal Investigator (RS) via email explaining the study and offering participation. The research team had no prior relationship with the participating APPs or SWs and the opportunity to participate was offered to all employees in each department via email and in their respective regularly scheduled team meetings. OACS participants were identified using purposive sampling by a review of MSK Survivorship clinic lists and contacted by a CRC via email with telephone follow-up to explain the study and offer participation. Lists of potentially eligible participants were based on patients with upcoming appointments in the survivorship clinics. CRCs used these lists and contacted participants in the order they were presented based on the soonest upcoming appointment.

Inclusion criteria for OACS were: (1) ≥65 years old; (2) English-speaking; and (3) History of cancer and currently being followed in the MSK Survivorship clinics. Patients in the survivorship clinics include all cancer types and patients tend to transition to these clinics for ongoing cancer surveillance 6 months to 5 years post active cancer treatment depending on their disease and disease course.

### Data collection

Demographic information was collected online via a short self-reported questionnaire and focus groups. The brief demographic questionnaire queried participants about sex, age, and health related variables. The 90-minute focus groups were implemented using Zoom videoconferencing. Only participants and study researchers were present for data collection and focus groups. Participants received $20.00 as compensation for participation.

We aimed to recruit 5–8 participants per focus group to foster discussion while ensuring all participants had time to contribute as they wished, and each focus group was conducted in a single session. Focus groups were held separately based on participant group (i.e., 1 SW group, 1 APP group, and 2 OACS groups). The semi-structured focus group guide was developed by the study team and included two parts. The first focused on querying participants about their experiences referring and connecting to mental health services during cancer survivorship with a focus on barriers and facilitators. The second half of the interview solicited feedback on a specific intervention (these results will be reported elsewhere). The interview guides for providers (i.e., APPs and SWs) was slightly different than that for OACS (see supplemental files for interview guides) Participants were recruited until thematic saturation was achieved (i.e., based on team meetings and discussion of transcripts) [25]. All interviews were audio-recorded and transcribed verbatim for analysis.

### Data analysis

Descriptive statistics from the demographic questionnaire were generated in SPSS. Thematic content analysis (TCA) guided the generation and interpretation of major and minor study themes from the focus groups [26–30]. NVivo version 14 (Lumivero, Denver, CO) was used for analyzing focus group transcripts. Use of a qualitative data analysis software assists in data management and organization, while retaining the researchers as analytic agents [31]. An interpretivist approach was employed, emphasizing context and subjective experience in meaning-making. First, the coding team (RS, DJ, EP, LW) independently reviewed one APP focus group transcript for data familiarization and code development via a primarily inductive approach. Codes were consolidated and defined in a group consensus meeting. Then, this transcript was re-coded by each coding team member using the updated codebook. Coding discrepancies were resolved in a second consensus meeting. This process was repeated for social work and OACS focus group transcripts, with the coding team individually coding each transcript and then meeting to reach consensus on code application and development of new codes (i.e., all four coders reviewed all four transcripts). Upon completion of preliminary coding, the first author synthesized coded data from across all participant groups, reviewing code reports, consolidating and categorizing codes, and identifying themes across focus groups. Group-specific patterns were also examined. In the final stage, results were presented to the coding team and the QMS for consensus and refinement.

## Results

Participant demographic characteristics are described in Tables 1 and 2 with a total sample of 22 OACS and providers (i.e., 9 OACS, 7 APPs, and 6 SWs). For the OACS groups, there were slightly more females than males (56%; *n* = 5) with a mean sample age of 74 (range: 66–85). Providers were also predominately female (92%, *n* = 12) and 69% (*n* = 9) had been working in oncology for over ten years. Three major conceptual themes were identified: (1) Care pathway characteristics; (2) Patient-level characteristics; (3) Perspectives on telehealth. These overarching themes included sub-themes of barriers and facilitators to connecting OACS to mental health services. Barriers and thematically related facilitators are discussed together below. Additional illustrative quotations and associated key concepts are included in Table 3.

**Table 1:** Patient demographic characteristics (*n* = 9)

Characteristic	*n* (%)	Characteristic	*n* (%)
Gender		Relationship Status	
Male	4 (44%)	Single	1 (11%)
Female	5 (56%)	Married/Partnered	5 (56%)
Race		Divorced/Separated	2 (22%)
White/Caucasian	9 (100%)	Other	1 (11%)
Education		Cancer Type	
Trade/Vocational School	1 (11%)	Breast	5 (56%)
Some Post-Graduate	1 (11%)	Blood	1 (11%)
Completed Post-Graduate	7 (78%)	Colon/Rectum	1 (11%)
Age		Lung	1 (11%)
M (SD)	74.3 (6.6)	Lung & Prostate	1 (11%)
Range	66–85	History of psychiatric treatment (any): Yes	6 (67%)
Self-Reported Health			
Good	3 (33%)		
Very Good	6 (67%)		

**Table 2:** Provider demographic characteristics (*n* = 13)

Characteristic	*n* (%)	Characteristic	*n* (%)
Gender		Position at MSK	
Male	1 (8)	Social Work	6 (46)
Female	12 (92)	Survivorship	7 (54)
Race		Years in Oncology	
White/Caucasian	10 (77)	1–5	4 (31)
Asian	1 (8)	10+	9 (69)
Other	2 (15)	Years in Geriatrics	
Hispanic: Yes	2 (15)	1–5	2 (15)
		6–10	2 (15)
		10+	9 (69)

**Table 3:** Qualitative themes and illustrative quotations

Concept	Illustrative quotations
Theme 1. Care Pathway Characteristics	
Introducing mental health services upon transition off of active treatment	“Oftentimes patients don’t even absorb or take in their entire experience from when they were diagnosed to their treatment until they really get to the first survivorship visit. And then, it’s like that time to kind of pause and really take in all of what that whole experience was. And so, that’s often a very emotional visit for my patients and then a good kind of segue into discussing referrals.” (APP Focus Group) “Perhaps their first post-treatment appointment with the nurse practitioner or oncologist—as they’re trying to segue back into their life, I think that might be the right time.” (Patient Focus Group 2)
Benefit of early depression screening and normalizing language	“I just try to be aware, particularly how we do these surveys. And if they score high on the depression screen, the way that I introduce to try to offer the supportive counseling or help in a way that they don’t automatically reject.” (APP Focus Group) “I think people are really receptive when I’m like, “they can just give you a call and you can see what’s out there, but it’s not binding to seeing a therapist or anything or talking to somebody. It’s just someone to check in and let you know what resources are available here.” (APP Focus Group) “I try to not phrase it in the way of formally talking about psychiatric care or a psychologist. I kind of just phrase it like everybody needs somebody to talk to.” (APP Focus Group)
Lack of available providers	“Let alone find someone who takes Medicare, but to find someone who takes Medicare that’s available, it’s 100% impossible.” (SW Focus Group) “Most recently just given the state of the world and the pandemic when I’ve tried to even refer to social work, they’ve actually said that they’ve been inundated with referrals and so just gave me the resources of how to search for local therapists for patients to hand off to them.” (APP Focus Group)
Burden of multi-step coordination on patients	“And I find when patients are severely depressed, sometimes it’s very hard and not always a realistic expectation to ask them to do the bulk of the legwork around finding a provider and establishing a provider, just because of the state of depression and the symptoms that it can leave patients with.” (APP Focus Group) “I think sometimes people are just so stuck in the like cycle of living with a chronic illness that it’s just hard to have another thing to do to reach out and try to schedule a mental health appointment and it just feels like too much.” (SW Focus Group)
Importance of warm handoff/known provider	“I do think that we take ownership of really being the ones to initiate conversations and treatments. But it’s frustrating because we don’t really have many resources and many options for what we can offer. So, I think I like that we’re the ones that are able to do that, and I think they really do put their trust in us and our institution.” (APP Focus Group) “The chemo nurses—not that the oncologist didn’t spend time with you, but the nurses seemed to be easier to speak with. And they would comfort you while you were going through the chemo, and you just felt more comfortable asking different kinds of questions with them and discussing certain problems that you might be embarrassed to talk to the oncologist about.” (Patient Focus Group 1) “I would say because when you visit the doctor, the oncologists, there is—it’s very pressing. You know, there’s a lot of stress and so on. With my nurse practitioners, I felt like there was much more opportunity to just ask anything that was on my mind, whether it was medical or not, obviously medical, more mental. That seemed like a better place for me.” (Patient Focus Group 2)
Provider overwhelm	“I don’t think I’ve been very successful, actually, in being helpful to the older depressed population. And I think it’s because I’m sort of feeling their sense of overwhelmingness and things like mobility issues and how do they get out, how do they do anything? […] And I don’t quite know in the space of a visit, how do I make a crack in that and what would I offer them? I just wanted to sort of confide that I don’t think I’ve been particularly good at it.” (APP Focus Group)
Theme 2. Patient-level characteristics	
Limiting attitudes and beliefs	“I know with some patients, they’ll be like, well, what are they [mental health providers] going to do? They’re not going to change anything. My cancer situation isn’t going to be changed, so what can they really do for me?” (APP Focus Group)
Role of stigma	“I do think with especially older adults, that sometimes one barrier can be a stigma, just around seeing mental healthcare professionals […] I can think of a few older adult patients that I see who are afraid of losing their independence if they get help.” (APP Focus Group) “It took me a while because there’s always that stigma. Well, now I not only have cancer, but now I feel like I’m really depressed, and I need to see somebody. So, there’s that stigma there.” (Patient Focus Group 1) “Cancer was an excuse for me to get psychological help and then to go on and deal with other difficulties.” (Patient Focus Group 1)
Trust in the institution/trusted providers	“Patients who are in the system are understandably very attached to the system, so [they] really do not want to look outside of our system or they want to go to the Counseling Center or try to have more consistent support from social workers, even though in our roles we can’t be a therapist. So sometimes I find that difficult to help them think about moving outside of our system, especially if they get the majority of their healthcare here.” (SW Focus Group)
Information seeking and support groups	“When I was going through chemotherapy, I really wanted to reach out and hear other people’s experiences because I felt that it would give me more help in thinking that I could beat this, and it would make me feel a little bit stronger about going through each treatment.” (Patient Focus Group 1) “I found that listening—it was an hour and a half group—I’d come home, and I’d be so depressed from what went on in the group that it took me days to get over the group. So that didn’t prove so helpful.” (Patient Focus Group 2)
Theme 3. Perspectives on telehealth for OACS	
Benefit of seeing OACS in person	“I think that is also one of the downsides of telehealth, even though you physically can see them on a video, that you really can’t—when they at least walk into clinic, you can see them, just even how they’re dressed. Sometimes, you can get a sneak peek into their environment which sometimes can be helpful. But just kind of seeing their face isn’t as much as sort of seeing their body or their knees tremor.” (APP Focus Group)
Limited technology skills	“But when they hear that something is virtual, it tends to make them nervous and often the people that I’ve seen have said they’re not tech-savvy, and they’re not able to do something like that.” (SW Focus Group)
OACS endorsement of telehealth	“The convenience of not having to travel into the city is very helpful.” (Patient Focus Group 2) “Any medium should be used that is best suited for the individual, whether it’s teleconferencing, whether it’s Zoom, whether it’s email, whatever medium can be used should be used.” (Patient Focus Group 2)

### Theme 1. Care pathway characteristics

All participants reported difficulties along the full continuum of the care pathway, from referral to receipt of appropriate services. OACS reported feeling uncertainty around how to start the process of getting support. Both providers and OACS agreed that mental health services should be introduced early on, as patients are transitioning to survivorship care. Providers described the importance of leveraging depression screening at the first survivorship visit to facilitate referrals and discussion of mental health. Providers also noted the importance of normalizing support-seeking to mitigate any stigma or fears that OACS may have about acknowledging mood symptoms or the need for mental health treatment, and carefully tailoring language to avoid references to psychiatric care.

Lack of available providers was a persistent issue discussed by all participants. Providers felt challenged in being unable to refer patients in survivorship to mental health services within the institution and expressed a general lack of knowledge about where to refer OACS in the community. OACS concurred that finding a “good therapist” is difficult, especially one who understands the unique challenges of cancer survivorship or who takes Medicare. These challenges were further exacerbated by the COVID-19 pandemic, contributing to greater difficulty connecting OACS to mental health treatment. Providers shared that previous reliance on institutional social work services was no longer a feasible option due to increasing patient volume.

Similarly, multi-step care coordination (i.e., multiple steps between making a referral and a patient actually identifying and seeing a mental health clinician) could limit referral uptake. Providers noted the lack of opportunity for a smooth handoff to mental health providers and lack of integration with OACS’ primary care creates added burden on the patient, often resulting in lack of service initiation. In contrast, participants agreed that having a warm handoff (i.e., from survivorship care clinician to mental health provider) or a known provider introducing mental health services to OACS would increase the likelihood of referral uptake and treatment initiation. APPs noted they would be comfortable being the ones to introduce these services in survivorship and OACS agreed that nursing staff would be well suited for this role. They felt it would be a burden to oncologists to spend time discussing these concerns.

Overall, providers expressed feeling challenged to help OACS get connected to mental health services given these barriers and limitations, despite their commitment and enthusiasm to help their patients.

### Theme 2. Patient-level characteristics

Participant input converged on several concepts related to patient-level barriers to engaging in mental health treatment including attitudes towards and preferences for care, time, physical symptoms, stigma, and language and culture. The predominant facilitators of mental health treatment-seeking at the individual level were caregiver involvement, trust in the institution, and information-seeking.

Providers stated that patient attitudes, marked by a lack of interest, low motivation, and skepticism that mental health intervention can help in the context of cancer decreased the likelihood of OACS engaging in mental health care. OACS and providers had varied opinions on the role of stigma. Across participants, there were observations about how stigma impacts treatment-seeking, which sometimes connected to fears about the consequences of seeking treatment, such as loss of independence. However, OACS identified cancer as means to overcome stigma, “giving an excuse” for therapy.

Time, competing demands post-cancer, and prioritization of physical problems were also cited as factors influencing OACS’ engagement in mental health care. Participants described feeling overwhelmed searching for a provider, physical comorbidities such as mobility limitations, and fatigue as factors making initiation of mental health treatment particularly challenging. Providers noted that the presence of a caregiver enhanced the likelihood of OACS connecting to care, in part because of their encouragement and logistical help in getting the patient in touch with a provider or appointment.

OACS shared specific care preferences such as a desire to see mental health providers within their cancer center (i.e., trusted providers) and hesitation about trying psychiatric medication. Support groups were identified as a good opportunity to obtain information from peers about treatment options, managing side effects, and discussing providers. However, OACS were interested in individual counseling in the context of the limitations of support groups, such as sometimes finding them to be too upsetting or dissatisfying. Providers also noted that consideration of patient language and cultural preferences could be a challenge as they were sometimes unsure how to meet the potentially unique needs of diverse OACS seeking psychosocial support.

### Theme 3. Perspectives on telehealth for OACS

While telehealth was endorsed enthusiastically overall, providers expressed ambivalence about telehealth options for OACS in cancer survivorship, sharing specific concerns about its potential challenges to care in this population. Providers weighed concerns about not being able to see the full clinical picture of OACS via telehealth with the benefit of observing them in their homes. For example, APPs noted that via telehealth the clinicians can get a “sneak peak of their environment” but cannot see how they walk or how they’re dressed, limiting assessment. Some OACS may have limited technological skills and therefore be unable to take advantage of opportunities such as virtual support groups.

Despite this hesitation, providers did contend that having telehealth as an option overall increased OACS’ capacity to engage in care in survivorship. OACS overwhelmingly endorsed telehealth and virtual formats as an acceptable and feasible option to facilitate mental health treatment.

## Discussion

This study of multi-level stakeholders revealed the nuances and challenges of initiating mental health treatment for older adult cancer survivors (i.e., ≥ 65 years, post-cancer treatment). OACS, social workers, and APP perspectives all converged on three main themes with several barriers and facilitators embedded in each: (1) Care pathway characteristics; (2) Patient-level characteristics; (3) Perspectives on telehealth.

Results echo the care pathway challenges outside of cancer survivorship that often prevent older adults from receiving mental health care [32–34]. Specifically, it is widely recognized that many providers are not adequately trained to work with older adults [33]. In the context of cancer survivorship, this can be even more challenging when OACS feel that their providers do not fully understand cancer or its implications for their mental health. Developing readily disseminable interventions specifically for this population could help to attenuate provider and OACS fears about the adequacy of treatment for their unique clinical needs. Similarly, as described in our results, most mental health providers do not take Medicare (90% of Medicare beneficiaries are ≥ 65 years old) and therefore even if a provider is identified, many OACS cannot afford services [32, 34]. In the absence of Medicare policy changes that motivate provider participation, other areas of geriatric psychiatry have trained lay providers to deliver evidence-based interventions to overcome provider shortage barriers [35]. Examining the potential impact of these interventions for OACS could create additional, low-cost resources to address some of the care path concerns raised in this study.

Despite these challenges, our participants identified opportunities to develop standard practices to improve the introduction and likelihood of uptake of mental health services. For example, all groups felt that having a trusted nurse introduce services early on after depression screening in survivorship is an acceptable and perhaps more accessible opportunity (e.g., compared to an oncologist) for OACS to consider a mental health referral. Consistent with our past research, they also noted the importance of using non-stigmatizing language to introduce these services (e.g., avoiding terms such as “psychiatric” in favor of a “focus on support and what we can do to help you in this area,” or “everyone needs someone to talk to”) [36, 37]. This approach has the potential to help reduce fears that OACS may have about the implications of acknowledging depression or anxiety. Frontline clinicians can be trained to use alternative language when working with patients such as OACS who may be more susceptible to stigma around mental health treatment.

At the patient level, providers noted that OACS typically trust their cancer care team and are hesitant to see an outside provider even for mental health treatment. However, cancer centers are not equipped to treat the mental health of all cancer survivors. Participants also noted that some OACS may have such significant depressive symptoms that they do not have the energy or motivation to look for a mental health provider. Here, a “warm handoff” to a known provider in the community could overcome both the trust concerns and requirement of the OACS to take the lead on connecting to care. The “warm handoff” is a widely recognized approach to increasing the likelihood of receipt of services but is relatively understudied and could be the focus of future research in cancer survivorship and primary care, for example [38]. Also at the patient level, participants noted that OACS tend to prioritize physical concerns and minimize the potential benefit of mental health services in the context of cancer. This is another opportunity for frontline clinicians to provide psychoeducation or “coaching” to OACS to tactfully demystify these services and describe their potential benefits.

Finally, an emergent theme across groups was the benefit of telehealth for mental health services for OACS. Provider perspectives were mixed as providers worried that telehealth may lack some of the nuances of an in-person evaluation such as being able to see how a patient walks or their hygiene and personal care. However, they also noted the potential clinical benefit of observing OACS in their homes as a “sneak peak” into their environment. In contrast, OACS participants enthusiastically supported the use of telehealth to overcome the travel and time considerations associated with in-person mental health care. In the evolving telehealth landscape since the COVID-19 pandemic, best practices for the delivery of treatment to OACS via telehealth will continue to evolve to ensure the provision of quality care, and our results highlight its ability to increase access by overcoming the challenges of accessing in-person care (i.e., unless an in-person assessment is clinically indicated).

### Limitations

Limitations of the present study include its reliance on English-speaking OACS drawn from a single, well-resourced cancer center. Therefore, our results likely underestimate the challenges of connecting OACS to mental health treatment, as participants who speak other languages and/or have different cultural backgrounds and other community-based institutions may not have the same resources for screening and referral as the current institution. The challenges for other OACS subgroups and at other institutions may also be different. We also did not collect information about OACS’ socioeconomic status, cancer treatment type or timing, which likely influenced their perceptions and experiences. Additionally, we did not restrict our OACS sample to only participants who had a known history of mental health treatment or concerns. We opted to include broader perspectives by including all OACs, but our findings may miss some key perspectives without oversampling individuals with more lived experience of mental health concerns. Similarly, our sample included all OACs who were comfortable participating in focus group study via videoconferencing. Thus, conclusions about the acceptability of tele-mental health treatment should be interpreted with caution. Future research in this area should draw on perspectives from diverse clinical care settings with a wider range of OACS and provider perspectives represented. Additionally, we sought to balance results by sharing illustrative quotations from across focus groups (see Table 3), but as speakers within transcripts were de-identified (i.e., most did not state their participant ID before speaking), we cannot confirm that all quotes were drawn from unique participants. Finally, the present study does not allow for conclusions regarding the specific challenges faced by OACS relative to non-cancer peers or younger adult cancer survivors, but these nuances can be explored in future mixed methods research across the lifespan.

## Conclusions

Despite feeling a significant need and interest in referring, providers struggle to overcome systemic barriers to getting OACS the mental health care they need. Given the standard of care to screen for depression in cancer survivorship, older patients will continue to be identified as depressed with no clear recourse for intervention. Given these results, future research on how to mitigate barriers and improve existing care pathways to connect OACS to mental health treatment is warranted.

## Supplementary Information

Supplementary Material 1.

## Data Availability

The data generated during and/or analyzed during the current study are available from the corresponding author on reasonable request.
