# Supplementary material for: Multi-level stakeholder perspectives on barriers and facilitators to engaging older adult cancer survivors in mental health treatment
Source: BMC Cancer. 2026 Jul 3;26:1137. doi: 10.1186/s12885-026-16396-2 (PMC13602514; doi:10.1186/s12885-026-16396-2)
Supplement: Supplementary file 1 [file 12885_2026_16396_MOESM1_ESM.docx]

**Patient Interview Guide**

**We would like to get a sense of your thoughts about getting emotional support during cancer treatment and survivorship.**

- What types of services come to mind for you when you hear the term “mental health treatment?”
- Of these, which do you think you would be most likely to utilize if you felt you needed to?
- When you imagine accessing these services, what are the things that might get in the way of actually doing so (i.e., barriers)?
- What things might make it easier for you to utilize these services (i.e., facilitators)?
- How would you want these options presented to you (e.g., oncologist, social worker, other care team member, volunteer services, etc.)?

**Next, we would like to understand how you have managed to engage in activities or trying new things to enhance your emotional wellbeing since your cancer diagnosis.**

- How has having cancer influenced your daily routine and interest in hobbies or other pleasurable activities post cancer?

**Wrap-Up**

- Thank you so much for speaking with us today. Are there any other aspects of your experience that you would like the share, that we did not have the chance to discuss?

**Provider Interview Guide**

We would like to get a sense of your thoughts about engaging older cancer survivors in mental health treatment.

- What have your experiences been getting older adults to utilize mental health services, either with you or through other resources?
- What are the major barriers to connecting these patients to treatment in cancer survivorship?
  1. Individual level
  2. Institution level
  3. Systems level
- What things make it easier for you to get patients to utilize these services?
- “Who would be the best member of the care team to present these treatment options?” (e.g. oncologist, social worker, other care team member, volunteer services, etc.)?

Next, we would like you to understand your thoughts about treating depression in older adult cancer survivors.

- Are you aware of any therapy treatments developed specifically for older adult cancer survivors or older adults with cancer more broadly?
- Do you feel supported and well trained to help these patients with their unique developmental needs?

**Wrap-Up**

1. Thank you so much for speaking with us today. Are there any other aspects of your experience that you would like the share, that we did not have the chance to discuss?
